# Progestins Related to Progesterone and Testosterone Elicit Divergent Human Endometrial Transcriptomes and Biofunctions

**DOI:** 10.3390/ijms21072625

**Published:** 2020-04-09

**Authors:** Sahar Houshdaran, Joseph C. Chen, Júlia Vallvé-Juanico, Shayna Balayan, Kim Chi Vo, Karen Smith-McCune, Ruth M. Greenblatt, Juan C. Irwin, Linda C. Giudice

**Affiliations:** 1 Center for Reproductive Sciences, Department of Obstetrics, Gynecology and Reproductive Sciences, University of California, San Francisco, CA 94143, USA; sahar.houshdaran@ucsf.edu (S.H.); julia.vallvejuanico@ucsf.edu (J.V.-J.); sj.balayan@gmail.com (S.B.); kimchi.vo@ucsf.edu (K.C.V.); karen.mccune@ucsf.edu (K.S.-M.); juan.irwin@ucsf.edu (J.C.I.); 2 BioMarin Pharmaceuticals, San Rafael, CA 94901, USA; joseph.chen@bmrn.com; 3 Departments of Clinical Pharmacy, Medicine, Epidemiology and Biostatistics, University of California, San Francisco, CA 94143, USA; ruth.greenblatt@ucsf.edu

**Keywords:** progestins, endometrial stromal fibroblasts, inflammation, angiogenesis, transcriptome

## Abstract

Progestins are widely used for the treatment of gynecologic disorders and alone, or combined with an estrogen, are used as contraceptives. While their potencies, efficacies and side effects vary due to differences in structures, doses and routes of administration, little is known about their effects on the endometrial transcriptome in the presence or absence of estrogen. Herein, we assessed the transcriptome and pathways induced by progesterone (P_4_) and the three most commonly used synthetic progestins, medroxyprogesterone acetate (MPA), levonorgestrel (LNG), and norethindrone acetate (NETA), on human endometrial stromal fibroblasts (eSF), key players in endometrial physiology and reproductive success. While there were similar transcriptional responses, each progestin induced unique genes and biofunctions, consistent with their structural similarities to progesterone (P_4_ and MPA) or testosterone (LNG and NETA), involving cellular proliferation, migration and invasion. Addition of estradiol (E_2_) to each progestin influenced the number of differentially expressed genes and biofunctions in P_4_ and MPA, while LNG and NETA signatures were more independent of E_2_. Together, these data suggest different mechanisms of action for different progestins, with progestin-specific altered signatures when combined with E_2_. Further investigation is warranted for a personalized approach in different gynecologic disorders, for contraception, and minimizing side effects associated with their use.

## 1. Introduction

Progestins, compounds with progestational activity, include naturally occurring progesterone (P_4_) and a variety of synthetic steroids [1,2]. They are widely used for contraception and the treatment of endometriosis, endometrial hyperplasia, and endometrial cancer, and are also used for postmenopausal hormone therapy [1,2,3,4,5,6]. Synthetic steroids that are structurally related to progesterone, testosterone, and spironolactone constitute the main classes of progestins. Progestins are key constituents of many contraceptives and either alone, or in combination with estrogens, are currently used by >660 million women globally [6,7,8]. The contraceptive effects of synthetic progestins result from a mimicry of the actions of progesterone, including inhibition of ovulation and thickening of cervical mucus [6,9]. Additionally, they can counteract estrogen-driven endometrial proliferation in endometriosis and have applications in postmenopausal hormone therapy and endometrial hyperplasia and cancer [1,3,10,11,12,13]. Recently, progestins have been implicated in the increased risk of HIV-1 acquisition, perhaps by modulating the integrity and cellular functions of the female reproductive tract and impact on immune functions, HIV-1 replication, and the vaginal microbiome [14,15]. However, “all progestins are not equal” [1], as they have different structures that alter their affinities for the progesterone nuclear receptor (PR), elicit unique intracellular signaling pathways and exhibit different potencies, metabolism, pharmacokinetics, efficacy, side effects and off-target effects [2,9]. Patient hormonal status and progestin dose, route of administration, formulation, combination with or without an estrogen, and duration of use also contribute to different effects locally within the female reproductive tract (e.g., histology, vascular integrity) [12,16], in other PR-expressing tissues, such as breast [2,6], and systemically [6,9].

**Figure 1:**
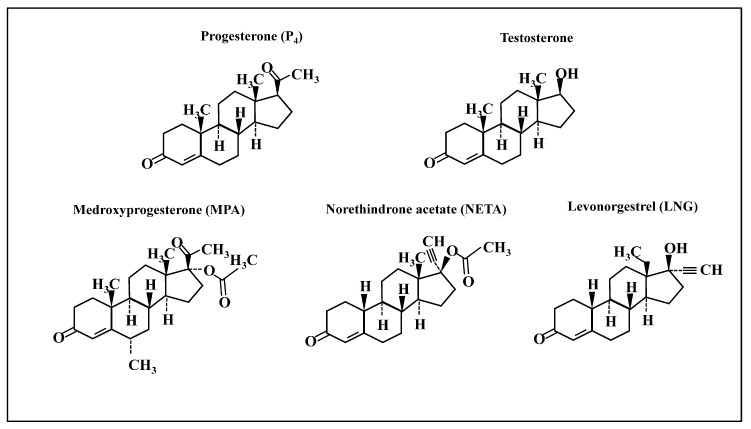
Chemical structures of progestins used in the study. Chemical structures of progesterone, testosterone, medroxyprogesterone acetate (MPA, a progestin structurally related to progesterone), and two progestins structurally related to testosterone: norethindrone acetate (NETA) and levonorgestrel (LNG).

The study of transcriptional regulation within endometrial tissue and cells in response to progestins (with and without estrogens) has received limited focus [17,18,19,20], although they offer insights into understanding the molecular and functional impact of these steroids. The current study examined the transcriptome and related biological and functional pathways of human endometrial stromal fibroblasts (eSF) in vitro, in response to different progestins with and without estradiol (E_2_). This cell type is a central effector of endometrial physiology, homeostasis, and pathophysiology across a woman’s lifespan, including regulating endometrial cycling, receptivity to an implanting embryo, and generation of the decidua of pregnancy [21,22]. Additionally, eSF exhibit abnormalities in endometriosis and polycystic ovary syndrome, and they respond in situ during contraceptive and post-menopause hormone therapy. Thus, the study of this cell type has promise to provide insights into why some progestins have more efficacy, consequential side effects (e.g., breakthrough bleeding) [6,9] and susceptibility to HIV acquisition [15]. Given the plethora of progestins and their diverse bioactivities, herein, we focused on the most commonly formulated progestins in use today: medroxyprogesterone acetate (MPA, structurally similar to progesterone (P_4_)) and levonorgestrel (LNG) and norethindrone acetate (NETA)—structurally related to testosterone (Figure 1).

In the current study, we found that these progestins commonly display anti-inflammatory and pro-angiogenic profiles, altered effects on extracellular matrix integrity and exhibited distinct transcriptomic profiles depending on their subclass—i.e., structurally related to progesterone versus testosterone and within progestin sub-classes. Notably, addition of estradiol (E_2_) moderated some of the effects depending on the progestin, indicating that structural differences in the progestins are important in gene regulation and interactions with other steroid hormones in the endometrium. 

## 2. Results

### 2.1. Progestins Structurally Related to Progesterone and Testosterone Induce Distinct Gene Expression Profiles

Gene expression profiles of eSF in response to each progestin versus vehicle control revealed that similar numbers of genes were differentially expressed in response to structurally related P_4_ and MPA; whereas, more than twice the number of genes were differentially expressed in response to LNG and NETA (Table 1). Moreover, LNG and NETA, structurally related to testosterone (Figure 1), affected similar numbers of differentially expressed genes (DEG) (Table 1 and Table 2). The top 30 DEG for each progestin treatment are presented in Table 2 (see Supplemental Table S1 for the full list of DEG for each progestin treatment).

Notably, in the response of eSF to all progestins, regardless of type, there were several classical progesterone-regulated genes, including secreted protein acidic and enriched in cysteine like 1 (*SPARCL1*), solute carrier family 7 member 8 (*SLC7A8*), olfactomedin (*OMD*), dikkopf 1 (*DKK1*), forkhead binding protein 5 (*FKBP5*), and interleukin 1 receptor (*IL-1R*) (Table 2) [23]. However, each progestin also differentially regulated unique genes compared to other progestins (full list in Supplemental Table S1), which were further altered in the presence of E_2_ (see below and Table 2). 

The unique and common molecular functions of each progestin effect on eSF were analyzed by Ingenuity Pathway Analysis^®^ (IPA) (Table 3).

The eSF response to P_4_ revealed that six molecular functions were decreased compared to vehicle control, including angiogenesis, endothelial cell development and proliferation, and cell viability and the secretion of lipid were increased. In response to MPA, cell viability, fibroblast differentiation, and tumor growth were decreased. Only one common function, decreased cell viability, was shared between P_4_ and MPA treatments of eSF (Table 3). When eSF cells were treated with progestins structurally similar to testosterone, there were considerably more altered molecular functions than in the P_4_ and MPA treated groups (Table 3). With LNG treatment of eSF, 37 molecular functions were regulated, including increased apoptosis and cell death and decreased cell movement, proliferation, migration, growth, and colony formation. Similar results were observed with NETA, wherein 41 molecular functions were regulated, including decreased cell movement, viability, survival, growth, invasion, proliferation, and migration. When comparing LNG and NETA treatment groups, 28 common molecular functions were observed, including decreased cell movement, cell migration, cell proliferation and cell viability, among others (Table 3).

### 2.2. Estrogen Enhances Progestin-Specific Effects on Gene Expression

The combined treatment of E_2_ with progestins resulted in higher numbers of differentially expressed genes compared to progestins alone (except for NETA), especially in the group structurally similar to progesterone (Table 1). Supplemental Table S1 and Table 2 contain the full gene lists and the top 15 up- and down-regulated genes in all groups, respectively. Of the 116 DEG in eSF treated with P_4_ versus vehicle control, 112 DEG (96.5%) were also differentially expressed in E_2_+P_4_ versus control. Of the 251 DEG expressed in eSF treated with MPA, 224 DEG (89.2%) were also expressed in the E_2_+MPA treatment group. In contrast, LNG and NETA exhibited similar effects (to each other) on changes in gene expression in the presence or absence of E_2_ (Table 1 and Table 2). Of the 553 DEG in eSF treated with LNG versus vehicle control, 511 DEG (92.4%) were also expressed in response to E_2_+LNG versus control. Of the 595 DEG resulting from NETA treatment, 502 DEG (84.3%) were also expressed in the E_2_+NETA treatment group.

**Figure 2:**
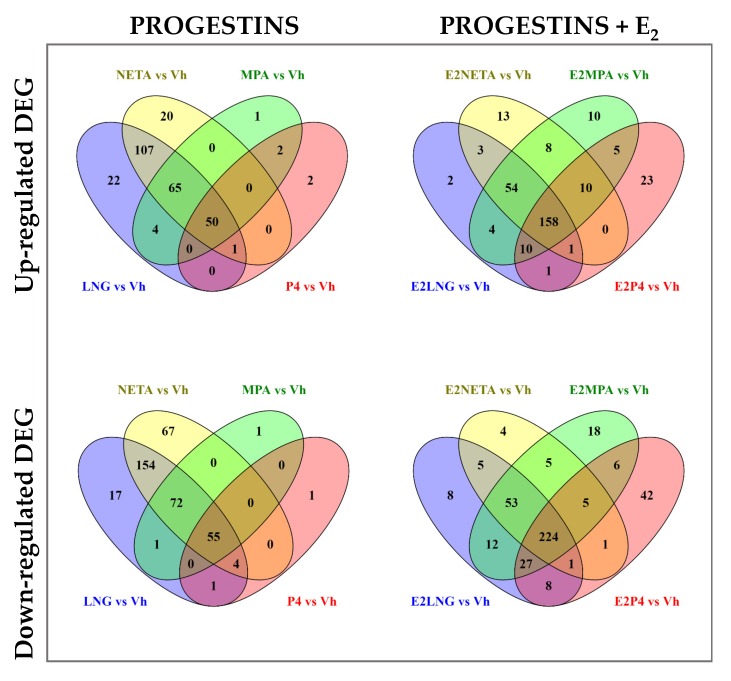
Venn diagram of differentially expressed genes among the four progestins alone or in combination with E_2_. Upper panel shows Venn diagrams of up-regulated DEG for each progestin versus Vehicle alone (left panel), or in combination with E_2_ (right panel). Lower panel shows Venn diagrams of down-regulated genes for each progestin versus vehicle alone (left panel), or in combination with E_2_ (right panel). Fold change (FC) ≥ 1.5 and Benjamini-Hochberg adjusted *p* < 0.05.

The Venn diagrams (Figure 2) show the number of upregulated and downregulated DEG in common and unique for each progestin treatment, with or without E_2_ versus vehicle control.

The P_4_ and MPA treatments upregulated 52 common genes, with only 2 genes uniquely expressed in P_4_ alone and 1 in MPA alone. LNG and NETA resulted in the upregulation of 223 common genes, with 22 and 20 genes uniquely upregulated in LNG or NETA, respectively. Overall, there were 50 genes commonly upregulated by all four progestins. The number of upregulated genes increased with the addition of E_2_, particularly in P_4_ and MPA, with 23 uniquely upregulated genes in the P_4_ and 10 genes in MPA. Altogether, there were 158 genes in common among all four progestins when combined with E_2_ (vs. 50 genes in the absence of E_2_). Similarly, the majority of downregulated genes in P_4_ treatment were in common with MPA (55 genes). LNG and NETA induced more DEG than MPA and P_4_, with 285 downregulated genes in common between LNG and NETA. In addition, the addition of E_2_ increased the number of downregulated genes—particularly in P_4_ and MPA. These data demonstrated that the addition of E_2_ affected up- and down-regulation and increased the total number of common DEG in all four treatments to 224 genes, compared with 55 downregulated genes in progestins without the addition of E_2_. The lists of unique genes and pathways with and without E_2_ treatment are shown in Table 4 and Supplemental Table S1.

### 2.3. Molecular Biofunctions

The numbers of molecular and cellular functions of DEG were also increased when E_2_ was added to progestin treatments of eSF, mainly for progestins structurally related to progesterone (Table 3). Twenty-one molecular functions were uniquely affected when E_2_ was added to P_4_, compared to 7 molecular functions affected by P_4_ alone (Table 3). Notably, E_2_ increased biofunctions involving the internalization of carbohydrate and the import of D-glucose and decreased cell movement, migration, invasion, and tumor cell colony formation, differentiation of fibroblasts, and gene transcription. When E_2_ was combined with MPA, the cellular functions that decreased included cell movement, invasion and migration. Common molecular functions in the MPA treatment with and without E_2_ included decreased fibroblast differentiation, and cell migration. Six shared molecular functions were observed between P_4_+E_2_ and MPA+E_2_, including decreased fibroblast differentiation and cell growth, invasion and migration (Table 3). In contrast, addition of E_2_ to the progestins structurally related to testosterone resulted in fewer affected molecular functions than without E_2_ (Table 3). Of the 36 molecular functions affected in LNG+E_2_, 29 of them were common to LNG alone, including increased apoptosis and necrosis, and decreased cell growth, viability, migration and proliferation, and colony formation. In NETA+E_2_, there were 31 cellular and molecular functions altered, of which 26 were common to NETA alone. These included decreased cell movement, growth, invasion and migration, and colony formation (Table 3). Twenty six molecular functions were common between LNG+E_2_ and NETA+E_2_ treatments, including decreased fibroblast differentiation and cell growth, invasion and migration (Table 3). Notably, eSF treatment with E_2_ alone resulted in decreased molecular functions of colony formation, proliferation of smooth muscle cells, migration of endothelial cells and leukocytes, and the inflammatory response (Table 3).

The unique versus common molecular biofunctions in the absence or presence of E_2_ are indicated in Table 3, based on the structurally related progestin groups.

#### 2.3.1. Unique Molecular Biofunctions

Molecular functions that were unique for each progestin, as well as the upregulated and downregulated genes in each function, in the absence and presence of E_2_, are presented in Table 4. Note that these were unique between progestin-alone groups (P_4_ vs. MPA vs. LNG vs. NETA) and unique between progestin plus E_2_ groups (P_4_+E_2_ vs. MPA+E_2_ vs. LNG+E_2_ vs. NETA+E_2_). Unique biofunctions for each progestin involved decreased angiogenesis and endothelial cell-related functions in P_4_, increased tumor cell migration and decreased tumor formation in MPA, increased cell death and necrosis in LNG, and decreased transcription, cell survival, vascular smooth muscle cell movement and migration in NETA. In the presence of E_2_, only P_4_+E_2_ and LNG+E_2_ (but not MPA+E_2_ and NETA+E_2_) showed unique biofunctions, which were different from those of unique P_4_ and LNG without E_2_ (Table 4).

#### 2.3.2. Common Molecular Biofunctions

Table 5 reflects the molecular biofunctions commonly shared among all four progestin treatments with and without E_2_, as well as the genes involved in each biofunction based on progestin treatments. In the absence of E_2_ (progestin alone treatments), only one molecular function, decreased cell viability, was common among all progestin treatments (Table 5).

In contrast, with the addition of E_2_, six biological functions were common to all four treatments, including decreased fibroblast differentiation, growth of connective tissue, and the invasion and migration of cells and tumor cells (Table 5). Interestingly, the one common DEG in all biofunctions and in all groups, with or without E_2_, was the chemokine CCL2 (bolded in Table 5). Two DEG, CCL2 and IL-6, were common among all biofunctions and across all progestin treatment groups in the presence of E_2_.

### 2.4. Assessment of Secreted Protein Levels of the Two Differentially Expressed Genes Common to All Treatment Groups

The protein products of CCL2 and IL-6 genes were assessed because they were the only differentially expressed genes common to all treatment groups, including progestins alone or combined with E_2_. Moreover, vascular endothelia growth factor A (VEGFA) was assessed due to its important role in angiogenesis and as it was found to be downregulated in five of the eight studied conditions.

**Figure 3:**
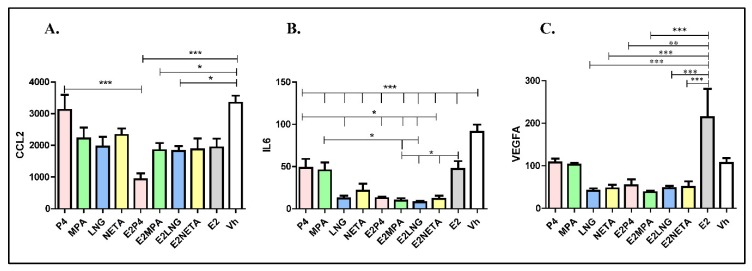
Concentrations of secreted CCL2, IL-6 and VEGFA in media conditioned by eSF after progestin and progestin plus E_2_ treatments. (**A**) Secreted CCL2. (**B**) Secreted IL-6. (**C**) Secreted VEGFA. In all cases, the figure shows the “amount” of secreted protein for each progestin and each progestin, plus E_2_ adjusted by cell number and total media. Pink-colored bars: progesterone (P_4_); green-colored bars: medroxyprogesterone acetate (MPA); blue-colored bars: levonorgestrel (LNG); yellow-colored bars: norethindrone acetate (NETA); gray-colored bars: E_2_ alone; white bars: vehicle. Error bars indicated SD; * *p* < 0.05, ** *p* < 0.01, *** *p* < 0.001.

Concentrations of secreted CCL2 and IL-6 in media conditioned by eSF after progestin and progestin plus E_2_ treatments were determined (Figure 3).

All progestins, with or without E_2_, decreased secreted CCL2, IL-6 and VEGFA protein levels compared to vehicle control (Figure 3A), consistent with the gene expression data (Table 6). 

The addition of E_2_ did not significantly alter the progestin inhibitory effect on CCL2 protein levels, except when combined with P_4_, resulting in a marked reduction of secreted CCL2 (Figure 3A). All progestins decreased IL-6 levels, with P_4_ and MPA having the least inhibitory effect (*p* < 0.05), and combined treatment with E_2_ further attenuated this effect (Figure 3B). E_2_ alone stimulates VEGFA, but progestins, alone or combined with E2, reduce its secretion (Figure 3C).

## 3. Discussion

The endometrium in natural cycles responds in a programmed fashion to E_2_ by induced cell proliferation, followed by P_4_-induced epithelial secretory transformation and stromal fibroblast decidualization, preparing for pregnancy. In non-conception cycles, it sheds and regenerates anew from epithelial and mesenchymal progenitors [24]. Normal endometrial homeostasis for growth, differentiation, desquamation, and regeneration revolves around appropriate cellular hormonal responses and paracrine interactions among the various cell types. Comprising this dynamic tissue are epithelial, endothelial, immune, vascular smooth muscle and stem cells, and stromal fibroblasts [25]. Progesterone promotes an epithelial-like phenotype of the latter, transforming them to master modulators of endometrial epithelial, vascular and immune function, acceptance of the conceptus, and controlled hemostasis during menses. Progestational agents share some, but not all, of native progesterone actions on eSF and are anticipated to have variable effects on this cell’s function in normal endometrial tissue and alternative effects not observed with P_4_ per se. Synthetic progestins are widely used for contraception, to treat endometriosis and endometrial cancer, and have been used in postmenopausal hormone therapy [1], and as a class, cause decidualization and atrophy of the endometrium [11,16]. A common side effect, often leading to their discontinuation, is abnormal uterine bleeding due to fragile endometrial vasculature [26] and overall altered signaling through the endometrial nuclear progesterone receptor (PR) [1,2]. The current study undertook, for the first time, an analysis of effects of synthetic progestins widely used in clinical practice on the human eSF transcriptional program, to identify each progestin’s effects and associated molecular functions relevant to normal and abnormal endometrial homeostasis. While we have identified the effects of these contraceptives on the eSF molecular phenotype, whether these differentially expressed genes are the direct or indirect targets of each progestins or reflect a transcriptional reprogramming in response to these hormone treatments are yet to be determined by time course and mechanistic analyses. 

### 3.1. Distinct Progestin-Induced Transcriptomes

In our comparison of the effects of four different and widely used progestins (P_4_, MPA, LNG and NETA) on the eSF transcriptome alone and in combination with E_2_, we found distinct differences between progestins structurally similar to progesterone (P_4_ and MPA) and those structurally similar to testosterone (NETA and LNG). As anticipated, the gene expression signatures of P_4_ and MPA treatments of eSF were more similar to each other and were different from signatures elicited by LNG and NETA, which were similar to each other, but also had unique transcriptomic patterns. 

In the response of eSF to all progestins, regardless of type, several classical progesterone-regulated genes were upregulated, including *SPARCL1, SLC7AB, OMD, DKK1, FKBP5*, and *IL-1R* and down-regulated were *CCL2, IL-6*, transforming growth factor β1 (*TGFβ1*), matrix metalloproteinase-3 (*MMP3*), and 17β-hydroxysteroid dehydrogenase 2 (*17βHSD2*) [23]. These genes are regulated via P_4_:PR (ligand:receptor) binding with PR-mediated signaling, and the stimulation or silencing of gene expression. That all four progestins had similar effects on transcription of classically P_4_-regulated genes underscores the importance of PR in signaling by all progestins studied. However, the various progestins stimulated and repressed unique genes, as well. The unique genes differentially regulated by each of these progestins involved vastly different biofunctions that could potentially have distinct effects on the endometrial function and progestin-induced endometrial changes. For example, P_4_ uniquely affects angiogenesis and endothelial cell development and proliferation, while unique genes in MPA affect cell migration, and unique LNG genes affect cell death and necrosis, with NETA affecting a wide range of biofunctions. This is likely due to different PR activation by each ligand leading to altered gene transcription, perhaps their binding to other steroid hormone receptors, for which these progestins have measurable affinity (see below), by altering the chromatin per se, and/or by non-genomic pathways [27]. An example of the latter is the progestin R5020 that at picomolar concentrations promotes the proliferation of rat endometrial stromal cells via ERK1-2 and AKT activation mediated by PR interaction with ER, resulting in the PR regulation of gene expression, independent of hormone receptor binding to specific genomic targets [28].

### 3.2. Estrogen Effect on Progestin-Induced Transcriptome

The addition of E_2_ considerably affected eSF gene expression profiles in response to P_4_ and MPA; whereas, when E_2_ was combined with LNG and NETA, only a modest effect on their gene expression signature profiles was observed. We note that, in this study, we have assessed the effects of estradiol and not ethinyl E_2_, a commonly used synthetic estrogen in oral contraceptives. This is because the focus of the current study is on progestins and their effects in the absence/presence of E_2_ priming/stimulation, due to the known effects of E_2_ on biological responses to progestins. We have used estradiol as prototype, which addresses this question without potential pharmacological confounders beyond physiologic estrogenic signaling. The potentially divergent actions of different estrogenic compounds warrant a different experimental design.

Diverse molecular mechanisms may contribute to the distinct transcriptional responses to the various progestins observed in the current study. Even subtle changes in progesterone structure or synthetic progestins with progestational activity and varied binding affinities for PR and other steroid hormone receptors (see below), and in the presence of E_2_ binding to its cognate nuclear receptor and by non-genomic pathways, can result in the altered regulation of gene expression. 

The effects observed herein likely derive from the unique structures of progestins with their additional chemical groups and conformations (Figure 1), that confer different binding affinities for cognate nuclear receptors [1]. For example, MPA, NETA and LNG have a greater affinity for PR than P_4_ (relative binding affinities of 115, and 150 and 50, respectively), and none of the synthetic progestins studied herein have an appreciable affinity for ER [29]. The binding affinity to the androgen receptor (AR) was reported to be higher for LNG and NETA than P_4_ [29]. However, in a steroid-receptor-deficient COS-1 cell line, with transiently expressing human AR; MPA, NETA, and P_4_ had a similar affinity to 5α-dihydrotestosterone (DHT) in binding to AR. However, NETA and MPA differed in inducing classical and AR-selective promoters [30]. Interestingly, it has been suggested that MPA displays greater glucocorticoid receptor (GR) agonist potency than P_4_ and NETA [31]. Moreover, MPA has immunosuppressive effects mediated by GR, as demonstrated by MPA repressing expression of IL-2 mRNA in normal human lymphocytes through GR [32].

It is important to note that the number of genomic regions containing exclusive hormone-specific response elements across the genome is limited, and many areas of the genome contain several response elements [33], which may explain how the combinatorial recruitment of ER with PR could alter target gene expression. Furthermore, progesterone-derived progestins binding to PR, MR and GR indicate that these progestins may use different mechanistic pathways from those of progestins structurally similar to testosterone, and subsequently have distinct differential combinatorial effects with ER.

### 3.3. Chemokines, Cytokines, and Angiogenic Factors

*IL-6* and *CCL2*, encoding major pro-inflammatory cytokines, were the only two genes in common in biofunctions of eSF treated with all progestins in the presence of E_2_. *CCL2* (also called monocyte chemoattractant protein-1 (*MCP-1*)) is a potent chemoattractant for monocytes and T cells [34], with lesser effects on basophils [35] and natural killer cells [36]. The *CCL2* gene is located in chromosome 17 and has a promoter region containing binding sites for AP-1 and NFKB, and is a major product of macrophages and other cell types [37]. Of note, Arici et al. reported an anti-inflammatory phenotype produced in eSF, when P_4_ or MPA were added to these cells in vitro, specifically with regard to down-regulation of the *CCL2* protein and mRNA [34]. Importantly, the effect of MPA on *CCL2* expression was reversed by the PR antagonist, RU486, demonstrating the PR-mediated regulation of this chemokine (25). In the current study, in addition to P_4_ and MPA, LNG and NETA, progestins structurally related to testosterone also down-regulated *CCL2*, in the absence or presence of E_2_. Of note, *CCL2* is highly expressed in peri-menstrual endometrium, when E_2_ levels are low but is down-regulated at ovulation, when E_2_ levels are high [38]. This regulation is consistent with the results obtained herein, demonstrating that its down-regulation is mediated by PR and ER.

Down-regulation of *CCL2* and *IL-6* in eSF treated with progestins and E_2_ suggests an anti-inflammatory environment in human endometrium. It is noteworthy that the downregulation of these two pro-inflammatory cytokines with all different progestin treatments is consistent with the known anti-inflammatory effects of progestins on human endometrium in vivo [11,16,39]. Early implantation is characterized by high levels of the pro-inflammatory T helper cells and cytokines such as TNFα, IL-6, and IL-8 [40,41], and the upregulation of pro-inflammatory cytokines positively correlates with IVF pregnancy outcomes [42]. Cytokines and chemokines such as IL-6 attract human trophoblast cells and therefore affect successful implantation [42], and the balance of pro- and anti-inflammatory cytokines and chemokines is required for proper endometrial tissue growth and remodeling [42,43]. Thus, downregulation of these cytokines has the potential to contribute to a sub-optimal environment for implantation and endometrial homeostasis.

CCL2 activates STAT1 signaling [44], and in mammals, the JAK/STAT signaling pathway is the principal pleiotropic cascade to transduce a wide array of cytokines and growth factors affecting pathways for cell differentiation, proliferation, migration and apoptosis [45]. Since the CCL2 receptor, CCR2, is present in human vascular smooth muscle cells (VSMCs) [46] and since STAT1 signaling induces VSMC proliferation [46], lowered *CCL2* expression in eSF in response to progestins may contribute to the decreased VSMC proliferation involved in endometrial blood vessel formation in the proliferative phase, their growth and maintenance in luteal phase endometrium and potentially vessel fragility—a known side effect causing breakthrough bleeding in women using these progestins [47,48].

Moreover, *VEGFA* mRNA and protein (Table 6, Figure 3C) were decreased in eSF treated with progestins. By extrapolation, progestins could inhibit blood vessel formation in endometrium, leading to an inhospitable environment for embryo implantation and breakthrough bleeding, the latter of which is commonly associated with this class of drug [6,9]. Breakthrough bleeding in long term progestin-only contraceptives users is due to a complex interplay of progestin effects on eSF and thrombin, tissue factor, IL-8, and VEGF, resulting in aberrant angiogenesis and the enhanced fragility of endometrial blood vessels [39]. While the current findings are consistent with the potential involvement of some of these progestin-regulated molecules in these processes, the precise mechanisms underlying the role of eSF in breakthrough bleeding in women on progestin-only preparations, as well as those with combined estrogen-progestin preparations, await further study. Moreover, whether these findings have implications for disorders with abnormal progesterone signaling, such as the endometrium of women with endometriosis [49] and polycystic ovarian syndrome [50] remains to be determined.

### 3.4. Strengths and Limitations of This Study

This is the first study to assess the effects of four commonly used progestins, alone or in combination with E_2_ on the genome-wide transcriptome of normal human endometrial stromal fibroblasts, adjusting for biopotency. The data support common and unique responses of this cell type to progesterone and the other progestins studied herein in the absence or presence of estradiol that are relevant to endometrial effects of these steroids widely used by women across the lifespan. The data provide insights into potential mechanisms underlying breakthrough bleeding and other side effects commonly encountered among women taking these medications. Of note, the current study was an in vitro analysis, and while progestin biopotencies were similar, local effects in vivo can be modulated by numerous confounders, including paracrine interactions among endometrial cellular components as well as different bioavailability, metabolism, and pharmacodynamics of these steroids, ultimately leading to composite effects at the cellular level as well as systemically. A limitation of our study is that the effects of progestins on other cell types of endometrium are yet to be determined. We aim to investigate the effect of these contraceptives in endometrial epithelial and immune cells, to gain better insights into their effects on human endometrium. Another limitation is that the concentrations used for the progestins were determined by dose-response experiments, to identify concentrations that elicit cellular responses. These are not similar to physiological concentrations and remain to be determined in women using these contraceptives.

In conclusion, in vitro progestin effects on eSF alone or in combination with E_2_, differ from one progestin to another, and those structurally similar to progesterone and testosterone more closely align with their respective group effects on eSF gene expression. Despite these differences, there were many genes and pathways shared among the different progestins. All four progestins (alone or in combination with E_2_) decreased the expression of *CCL2*, prominently involved in immuno-regulatory and inflammatory processes. The results overall indicate that progestins have an anti-inflammatory effect, enhanced by E_2_ on the eSF cell, contributing to an anti-inflammatory environment in the endometrium. Notably, eSF in women with endometriosis and polycystic ovarian syndrome show an abnormal response to P_4_, and whether different progestins display varied responses in these populations warrants further analysis, potentially leading to more personalized progestin therapies for women.

## 4. Materials and Methods

### 4.1. Samples

This study was approved by the Committee on Human Research of the University of California San Francisco (UCSF) (IRB: 10-02786). Samples were collected from the UCSF/NIH Human Endometrial Tissue and DNA Bank after written informed consent. For these studies, endometrial stromal fibroblasts (eSF) were isolated from endometrial biopsies and established in primary culture, as previously described [51]. Briefly, five eutopic endometrial biopsies were collected from women without any gynecological abnormalities. Each tissue biopsy was digested separately using collagenase and the tissue digests were then filtered using a 40micron cell strainer to separate the dissociated eSF from endometrial glands and undigested tissue. The eSF from the filtered through fraction were then established in monolayer primary culture and grown in medium supplemented with 10% charcoal-stripped fetal bovine serum (FBS), as previously described [51]. Thus, a separate eSF primary culture was established from each of the five different patient biopsies. For the hormone treatment experiments, sub-confluent primary cultures (approximately 75% confluent) were harvested and eSF seeded onto 24-well plates at 10^5^ cells/well and grown in 10% serum-supplemented medium as above until confluent. Confluent cultures were treated with the various steroid hormones in medium supplemented with 2% charcoal-stripped FBS, according to our previously described protocol [52]. In brief, cultures were pre-incubated in medium supplemented with 2% charcoal-stripped FBS without hormones for 24 h. After 24 h, the culture medium was replaced with fresh medium containing steroid hormones (E_2_, progestin, progestin plus E_2_) or vehicle control for 14 days, with media renewal every three days. Concentrations of E_2_ and P_4_ used were maximally effective concentrations, as shown before for eSF in vitro decidualization [51]. Other progestins were used at concentrations equipotent to that of P_4_, adjusted according to their reported transactivation biopotency relative to P_4_ [53]. Concentrations of progestins, alone or in combination with 10 nM E_2_, were: 1 µM P_4_, 54.7 nM LNG, 0.294 μM NETA, 6 nM MPA and ethanol vehicle control (Vh, (0.1% ethanol)). After 14 days of treatment, eSF conditioned media were collected for secreted cytokine analysis and cells were harvested by trypsinization, counted, and frozen at -80ºC until RNA extraction. Each sample was processed separately for RNA extraction and microarray, without pooling samples derived from different patient biopsies.

To ensure proper cellular responsiveness to P_4_, the conditioned media from each sample after 14 days of treatment was used for the analysis of IGFBP1 protein levels by ELISA, in duplicate and normalized to the cell count for each sample. IGFBP1 is a progesterone-responsive gene, and we found its increased protein level in all treatments, and the highest with E_2_+P_4_ treatment, as expected. See Supplementary Figure S1.

### 4.2. RNA Extraction and Whole Genome Microarrays

Cellular RNA was extracted using the NuceloSpin Tissue Kit (Marcherey-Nagel Inc, Bethlehem, PA), according to manufacturer’s recommendations and stored at -80ºC until use. RNA quality was assessed using Bioanalyzer 2100 (Agilent Technologies, Santa Clara, CA) and only samples with an RNA integrity number (RIN) higher than 7 were considered of high quality and used for further analysis by microarray. RNA samples were reverse transcribed to cDNA, followed by 2^nd^ strand DNA generation and cDNA generation. For microarray analysis, all hormone treatments for each of the five cell lines were prepared according to Affymetrix (Santa Clara, CA) specifications and hybridized to the Affymetrix Exon Expression Chip HuGene1_0-st-v1 gene array at the UCSF Genome Core facility, as previously described [49].

### 4.3. Whole Genome Microarray Data Analysis

A gene expression microarray data analysis was conducted using GeneSpring GX software (Agilent Technologies). The data from the raw CEL files were normalized together by RMA. The normalized data were then used to identify differentially expressed gene between different comparisons across the five biological replicates using ANOVA. To correct for multiple comparisons, we used the Benjamini–Hochberg correction, and significance was determined at *p*-value < 0.05 and a fold change (FC)) > ± 1.5. Venn diagrams were used to assess common DEG among the four progestins, using Venny 2.1.0 software [54]. Subsequently, all DEG lists for all comparisons were analyzed using IPA software (Ingenuity LLC, Portola Valley, CA) [55], to identify common and unique pathways among the different progestins. Pathways with a z-score > ± 2.00 were considered to be significant. 

### 4.4. Luminex Analysis

The validation of IL-6 (interleukin-6), CCL2 (C-C motif chemokine ligand 2 (monocyte chemotactic protein-1)) and VEGFA (vascular endothelia growth factor A) gene expression for secreted proteins was performed using multiplexed immunoassay analysis (Luminex, EMD Millipore, Burlington, MA, USA) of these analytes in eSF conditioned media in the various treatment groups. These analytes were selected because they were decreased in gene expression analyses and were common among all comparisons (IL-6 and CCL2) or importance in angiogenesis (VEGFA) (see Results). Conditioned media from treated eSF cells were centrifuged at 3000xg for five minutes to remove debris, and the supernatant was used for Luminex analysis, according to the manufacturer’s instructions. Briefly, conditioned media were incubated overnight in Luminex plates with antibody-coated fluorescent-dyed analyte-specific microspheres, and bound analytes were resuspended in sheath fluid and analyzed on a Bioplex bead sorter (Bio-Rad, Hercules, CA), adjusted by media volume and cell number. Statistical significance of concentration differences of analytes in specific comparisons was determined as *p* < 0.05 by ANOVA after Bonferroni’s multiple comparison correction, using GraphPad Prism V.5.

## Figures and Tables

**Table 1:** Number of DEG in comparisons of each progestin vs. vehicle in the absence or presence of estradiol.

	Comparison	Up-Regulated DEG (FC ≥ 1.5)	Down-Regulated DEG (FC ≥ 1.5)	Total DEG
Progestins without E_2_	LNG vs. Vh	249	304	553
	MPA vs. Vh	122	129	251
	NETA vs. Vh	243	352	595
	P_4_ vs. Vh	55	61	116
E_2_	E_2_ vs. Vh	88	76	164
Progestins + E_2_	E_2_LNG vs. Vh	233	338	571
	E_2_MPA vs. Vh	259	350	609
	E_2_NETA vs. Vh	247	298	545
	E_2_P_4_ vs. Vh	208	314	522

E_2_: estradiol; MPA: medroxyprogesterone acetate; LNG: levonorgestrel; NETA: norethindrone acetate; FC: fold change; DEG: differentially expressed genes.

**Table 2:** Select differentially expressed transcripts and genes in each progestin vs. vehicle without and with estradiol.

P_4_ vs. Vh		E_2_P_4_ vs. Vh		MPA vs. Vh		E_2_MPA vs. Vh		LNG vs. Vh		E_2_LNG vs. Vh		NETA vs. Vh		E_2_NETA vs. Vh	
Gene Symbol	FC	Gene Symbol	FC	Gene Symbol	FC	Gene Symbol	FC	Gene Symbol	FC	Gene Symbol	FC	Gene Symbol	FC	Gene Symbol	FC
SPARCL1	5.2	SPARCL1	41.8	SPARCL1	13.8	SPARCL1	32.6	SPARCL1	22.2	SPARCL1	23.6	SPARCL1	19.3	SPARCL1	24.3
SLC7A8	3.5	SLC7A8	15.6	SLC7A8	6.0	FKBP5	11.1	FKBP5	9.9	FKBP5	11.2	FKBP5	11.3	FKBP5	11.1
LCP1	3.4	GREB1	15.0	OMD	5.1	SLC7A8	10.4	SLC7A8	9.4	CNR1	8.8	SLC7A8	9.3	PARM1	9.1
FKBP5	2.9	LCP1	8.7	FKBP5	5.0	PARM1	10.2	CNR1	8.3	SLC7A8	8.7	CNR1	8.9	LCP1	8.0
GPX3	2.9	OMD	7.1	THSD7A	3.8	LCP1	9.3	LCP1	7.9	PARM1	8.6	PARM1	7.9	CNR1	7.9
IL1R1	2.7	FKBP5	7.1	IL1R1	3.5	OMD	8.9	PARM1	6.7	LCP1	8.1	LCP1	7.9	SLC7A8	7.8
OMD	2.6	CNR1	6.7	LCP1	3.4	DKK1	8.1	OMD	6.5	OMD	7.1	MAOB	7.7	OMD	6.7
DKK1	2.4	GPX3	6.6	GPX3	3.1	CNR1	8.1	MAOB	6.5	MAOB	6.8	OMD	7.6	MAOB	6.1
MT-TA	2.3	THSD7A	6.0	CNR1	3.0	MAOB	6.9	GREB1	5.8	DKK1	6.3	DKK1	6.1	DKK1	5.7
THSD7A	2.2	MAOB	5.6	CRISPLD2	2.9	CRYAB	5.3	DKK1	5.5	GREB1	5.1	CRYAB	5.4	ENPEP	5.6
LPAR1	2.1	DKK1	5.6	LAMA2	2.8	ULK4	5.2	ULK4	4.8	CRYAB	5.0	ENPEP	4.6	PLCL1	4.9
SPSB1	2.1	IL1R1	4.9	CRYAB	2.7	ENPEP	5.1	CRYAB	4.7	IL1R1	4.7	IL1R1	4.5	ULK4	4.9
SEMA3C	2.0	C10orf10	4.8	LPAR1	2.6	THSD7A	5.0	IL1R1	4.4	THSD7A	4.4	CRISPLD2	4.3	CRYAB	4.8
SERPINE1	2.0	ABHD5	4.7	MAOB	2.6	PLCL1	4.7	PLCL1	4.2	LPAR1	4.3	ALDH1A3	4.2	ALDH1A3	4.7
APOD	2.0	IMPA2	4.6	ABCC9	2.5	GREB1	4.6	LPAR1	4.1	ALDH1A3	4.3	PLCL1	4.2	ITPR1	4.4
TOX	−1.8	HSD17B2	−3.8	ETV1	−2.7	DACH1	−4.4	TNFRSF19	−4.4	DACH1	−4.2	TNFRSF19	−4.3	CLDN11	−4.2
ARHGAP29	−1.8	LYPD1	−4.1	NDNF	−2.7	CD34	−4.4	CHRM2	−4.4	MXRA5	−4.5	CD34	−4.3	CD34	−4.2
CXCL12	−1.9	NCKAP5	−4.2	FJX1	−2.8	TOX	−4.5	CD34	−4.4	TGFBI	−4.5	F2RL2	−4.5	TNFRSF19	−4.4
DACH1	−2.0	CHRM2	−4.2	HPSE2	−3.0	TGFBI	−5.1	LYPD1	−4.8	TNFRSF19	−4.6	TOX	−4.7	LYPD1	−4.6
GUCY1A3	−2.0	FGF7	−4.4	HSD17B2	−3.1	LYPD1	−5.4	PRELP	−5.1	LYPD1	−4.7	TGFBI	−4.8	TOX	−4.7
TNFRSF19	−2.0	CD34	−4.5	NCKAP5	−3.1	GBP4	−5.6	GBP4	−5.2	NCKAP5	−6.1	FJX1	−4.9	GBP4	−4.7
ITGA8	−2.2	EGR2	−4.8	CHRM2	−3.2	CCL2	−6.0	TGFBI	−5.2	PRELP	−6.1	NDNF	−4.9	NCKAP5	−5.1
CHRM2	−2.3	MXRA5	−5.0	GBP4	−3.2	NCKAP5	−6.3	NCKAP5	−5.2	ETV1	−6.2	NCKAP5	−5.0	CCL2	−5.1
ETV1	−2.3	NDNF	−5.0	SFRP1	−3.4	PRELP	−6.3	NDNF	−5.6	CCL2	−6.2	GBP4	−5.1	NDNF	−5.4
LYPD1	−2.4	TGFBI	−5.4	CST1	−3.4	NDNF	−6.6	FJX1	−5.9	GBP4	−6.4	PRELP	−5.7	PRELP	−5.4
GBP4	−2.4	ETV1	−5.7	LYPD1	−3.5	SFRP1	−6.8	CCL2	−6.4	NDNF	−6.5	CCL2	−6.2	SFRP1	−5.5
NDNF	−2.5	GBP4	−6.3	MMP3	−3.6	FJX1	−7.7	ETV1	−6.5	FJX1	−6.8	ETV1	−6.4	ETV1	−6.0
KRT19	−2.6	CXCL12	−7.2	KRT19	−3.7	ETV1	−8.0	SFRP1	−6.8	SFRP1	−6.8	SFRP1	−6.4	FJX1	−6.3
SFRP1	−2.6	CCL2	−7.4	CD34	−3.8	HSD17B2	−8.6	HSD17B2	−7.1	MMP3	−8.1	MMP3	−6.5	MMP3	−6.8
CD34	−2.9	SFRP1	−8.4	CCL2	−4.8	MMP3	−9.0	MMP3	−7.5	HSD17B2	−9.5	HSD17B2	−7.0	HSD17B2	−7.7

E_2_: estradiol; P_4_: progesterone; MPA: medroxyprogesterone acetate; LNG: levonorgestrel; NETA: norethindrone acetate; Vh: vehicle; FC: fold change.

**Table 3:** Common and unique molecular functions of progesterone and testosterone structurally related progestins in the absence or presence of E_2_.

	Progestins vs. Vh & Predicted Activation		Progestins + E_2_ vs. Vh & Predicted Activation		E_2_ vs. Vh & Predicted Activation	
	Molecular functions	z ≥ 2	Molecular functions	z ≥ 2	Molecular functions	z ≥ 2
**P4**	Angiogenesis	↓	Cell movement of epithelial cell lines	↓	Colony formation	↓
	Binding of endothelial cells	↓	Cell movement of tumor cell lines	↓	Proliferation of smooth muscle cells	↓
	Cell viability	↓	Chemotaxis	↓	Inflammatory response	↓
	Cell viability of tumor cell lines ^+^	↓	Colony formation of tumor cells	↓	Cell movement of blood cells	↓
	Endothelial cell development	↓	Cytostasis of tumor cell lines	↓	Cell movement of endothelial cells	↓
	Proliferation of endothelial cells	↓	Differentiation of fibroblasts ^++^	↓	Migration of endothelial cells	↓
	Secretion of lipid	↑	Formation of skin	↓	Leukocyte migration	↓
			Growth of connective tissue ^++^	↓	Response of tumor cell lines	↓
			Homing of cells	↓	Quantity of Ca2 +	↓
			Import of D-glucose	↑		
			Internalization of carbohydrate	↑		
			Invasion of cells ^++^	↓		
			Invasion of tumor cell lines ^++^	↓		
			Migration of breast cancer cell lines ^++^	↓		
			Migration of cells	↓		
			Migration of colorectal cancer cell lines	↓		
			Migration of tumor cell lines ^++^	↓		
			Proliferation of connective tissue cells	↓		
			Proliferation of lung cancer cell lines	↓		
			Proliferation of smooth muscle cells	↓		
			Transcription	↓		
**MPA**	Cell viability of tumor cell lines ^+^	↓	Cell movement of carcinoma cell lines	↓		
	**Differentiation of fibroblasts**	↓	Cell movement of sarcoma cell lines	↓		
	Growth of tumor	↓	Colony formation	↓		
	Import of D-glucose	↑	Colony formation of cells	↓		
	**Migration of sarcoma cell lines**	↓	**Differentiation of fibroblasts ^++^**	↓		
	Migration of tumor cells	↑	Growth of connective tissue ^++^	↓		
	Non-melanoma solid tumor	↓	Invasion of cells ++	↓		
	**Sphere formation of tumor cell lines**	↓	Invasion of tumor cell lines ^++^	↓		
			Migration of breast cancer cell lines ^++^	↓		
			**Migration of sarcoma cell lines**	↓		
			Migration of tumor cell lines ^++^	↓		
			**Sphere formation of tumor cell lines**	↓		
**LNG**	Adhesion of lymphoma cell lines	↓	Activation of DNA endogenous promoter	↓		
	**Apoptosis**	↑	**Apoptosis**	↑		
	Binding of lymphoma cell lines *	↓	Binding of lymphoma cell lines **	↓		
	**Cell death**	↑	Cell death **	↑		
	**Cell death of tumor cell lines**	↑	**Cell death of tumor cell lines**	↑		
	Cell movement of carcinoma cell lines *	↓	Cell movement of carcinoma cell lines **	↓		
	Cell movement of epithelial cell lines *	↓	Cell movement of sarcoma cell lines **	↓		
	Cell movement of sarcoma cell lines *	↓	Cell movement of tumor cell lines **	↓		
	Cell movement of tumor cell lines *	↓	Cell proliferation of carcinoma cell lines **	↓		
	Cell proliferation of carcinoma cell lines *	↓	Cell viability of tumor cell lines **	↓		
	Cell viability of tumor cell lines *	↓	Chemotaxis **	↓		
	Chemotaxis *	↓	Colony formation **	↓		
	Colony formation *	↓	Colony formation of cells **	↓		
	Colony formation of cells *	↓	Colony formation of tumor cells **	↓		
	Colony formation of tumor cell lines	↓	Differentiation of fibroblasts **	↓		
	Colony formation of tumor cells *	↓	Growth of connective tissue **	↓		
	Differentiation of fibroblasts *	↓	Growth of malignant tumor **	↓		
	Growth of connective tissue *	↓	Growth of tumor **	↓		
	Growth of malignant tumor *	↓	Homing of cells **	↓		
	Growth of tumor *	↓	Invasion of cells **	↓		
	Homing of cells *	↓	Invasion of tumor cell lines **	↓		
	Invasion of cells *	↓	Microtubule dynamics	↓		
	Invasion of tumor cell lines *	↓	Migration of breast cancer cell lines **	↓		
	Migration of breast cancer cell lines *	↓	Migration of cells **	↓		
	Migration of cells *	↓	Migration of sarcoma cell lines **	↓		
	Migration of colorectal cancer cell lines	↓	Migration of tumor cell lines **	↓		
	Migration of prostate cancer cell lines *	↓	Necrosis **	↑		
	Migration of sarcoma cell lines *	↓	Organization of cytoplasm	↓		
	Migration of tumor cell lines *	↓	Organization of cytoskeleton	↓		
	**Necrosis**	↑	Proliferation of connective tissue cells **	↓		
	Phosphorylation of L-tyrosine	↓	Proliferation of lung cancer cell lines	↓		
	Proliferation of cancer cells	↓	**Proliferation of smooth muscle cells**	↓		
	Proliferation of connective tissue cells *	↓	↓	↓		
	Proliferation of smooth muscle cells *	↓	Sphere formation of tumor cell lines **	↓		
	Proliferation of tumor cells *	↓	Transcription	↓		
	Rearrangement of cytoskeleton *	↓	Transcription of RNA	↓		
	Sphere formation of tumor cell lines *	↓				
**NETA**	Binding of lymphoma cell lines *	↓	Adhesion of lymphoma cell lines	↓		
	Cell movement of carcinoma cell lines *	↓	Binding of lymphoma cell lines **	↓		
	Cell movement of epithelial cell lines *	↓	Cell death **	↑		
	Cell movement of muscle cells	↓	Cell movement of carcinoma cell lines **	↓		
	Cell movement of sarcoma cell lines *	↓	**Cell movement of epithelial cell lines**	↓		
	**Cell movement of smooth muscle cells**	↓	Cell movement of sarcoma cell lines **	↓		
	Cell movement of tumor cell lines *	↓	**Cell movement of smooth muscle cells**	↓		
	Cell proliferation of carcinoma cell lines *	↓	Cell movement of tumor cell lines **	↓		
	Cell survival	↓	Cell proliferation of carcinoma cell lines **	↓		
	Cell viability	↓	Cell viability of tumor cell lines **	↓		
	Cell viability of tumor cell lines *	↓	Chemotaxis **	↓		
	Chemotaxis *	↓	Colony formation **	↓		
	Colony formation *	↓	Colony formation of cells **	↓		
	Colony formation of cells *	↓	Colony formation of tumor cell lines	↓		
	Colony formation of tumor cells *	↓	Colony formation of tumor cells **	↓		
	Cytostasis of tumor cell lines	↓	Differentiation of fibroblasts **	↓		
	Differentiation of fibroblasts *	↓	Growth of connective tissue **	↓		
	Growth of connective tissue *	↓	Growth of malignant tumor **	↓		
	Growth of malignant tumor *	↓	Growth of tumor **	↓		
	Growth of tumor *	↓	Homing of cells **	↓		
	Homing of cells *	↓	Invasion of cells **	↓		
	Import of D-glucose	↑	Invasion of tumor cell lines **	↓		
	Invasion of cells *	↓	Migration of breast cancer cell lines **	↓		
	Invasion of tumor cell lines *	↓	Migration of cells **	↓		
	Microtubule dynamics	↓	Migration of sarcoma cell lines **	↓		
	Migration of breast cancer cell lines *	↓	Migration of tumor cell lines **	↓		
	Migration of carcinoma cell lines	↓	Necrosis **	↑		
	Migration of cells *	↓	Proliferation of cancer cells	↓		
	Migration of prostate cancer cell lines *	↓	Proliferation of connective tissue cells **	↓		
	Migration of sarcoma cell lines *	↓	Proliferation of tumor cells **	↓		
	Migration of smooth muscle cells	↓	Sphere formation of tumor cell lines **	↓		
	Migration of tumor cell lines *	↓				
	Migration of vascular smooth muscle cells	↓				
	Proliferation of connective tissue cells *	↓				
	Proliferation of lung cancer cell lines	↓				
	Proliferation of smooth muscle cells *	↓				
	Proliferation of tumor cells *	↓				
	Rearrangement of cytoskeleton *	↓				
	Sphere formation of tumor cell lines *	↓				
	Transcription	↓				
	Transcription of RNA	↓				

E_2_: estradiol; P_4_: progesterone; MPA: medroxyprogesterone acetate; LNG: levonorgestrel; NETA: norethindrone acetate; Vh: vehicle; ↓, Decreased; ↑, Increased; +: Common between P4 and MPA; *: Common between LNG and NETA; ++: Common between P4+E2 and MPA+E2; **: Common between LNG+E2 and NETA+E2; **Bold:** Common in each progestin without or with E2.

**Table 4:** Unique molecular functions and genes of each progestin without or with addition of E_2_.

	Unique P_4_		Unique P_4+_E_2_		Unique MPA		Unique MPA+E_2_		Unique LNG		Unique LNG+E_2_		Unique NETA		Unique NETA+E_2_	
	Molecular functions	z ≥ 2	Molecular functions	z ≥ 2	Molecular functions	z ≥ 2	Molecular functions	z ≥ 2	Molecular functions	z ≥ 2	Molecular functions	z ≥ 2	Molecular functions	z ≥ 2	Molecular functions	z ≥ 2
	Angiogenesis	↓	Formation of skin	↓	Migration of tumor cells	↑			Cell death	↑	Activation of endogenous promoter	↓	Cell movement of muscle cells	↓		
	Binding of endothelial cells	↓	Import of D-glucose	↑	Non-melanoma solid tumor	↓			Necrosis	↑	Organization of cytoplasm	↓	Cell survival	↓		
	Endothelial cell development	↓	Internalization of carbohydrate	↑					Phosphorylation of L-tyrosine	↓	Organization of cytoskeleton	↓	Migration of carcinoma cell lines	↓		
	Proliferation of endothelial cells	↓											Migration of smooth muscle cells	↓		
	Secretion of lipid	↑											Migration of vascular smooth muscle cells	↓		
													Proliferation of lung cancer cell lines	↓		
													Transcription	↓		
	**Unique Up- and Down-Regulated Genes**															
	**2 loci exclusively in “P4 vs. Vh”**		**23 loci exclusively in “E2P4 vs. Vh”**		**1 loci exclusively in “MPA vs. Vh”**		**10 loci exclusively in “E2MPA vs. Vh”**		**22 loci exclusively in “LNG vs. Vh”**		**2 loci exclusively in “E2LNG vs. Vh”**		**20 loci exclusively in “NETA vs. Vh”**		**13 loci exclusively in “E2NETA vs. Vh”**	
**Up-regulated Genes**	ABCA1, ABCG1		IRS1, PTS, ITGB1BP1, LAMA3, DPH3, MRAS, JMY, MFSD5, PPP2CB, CCT5, B3GALT4, DTNA, HSD17B11, TUBB2A, CNTN3, C20orf194, CORIN, PRKCDBP, KIF13A, UNG, SLC22A23, FMN1, POLD4		CH25H		P2RY1, SETMAR, HSPB1, BLVRB, RN7SKP283, ZDHHC7, TMEM120A, CDC25B, TMED10, PDE1A		TCEAL1|TCEAL3, OAT, TCEAL6, CDC42SE2, FAM199X, TRIM63, STK3, REV3L, SIDT2, GADD45A, PDE8A, NXPE3, PGR, GM2A, ATL3, TUBB2A, MORF4L2, SETMAR, TCEAL4, ATL1, PXK, PRTG		ARHGAP10, SMPD2		SNORD13, ABLIM1, GALNT10, TMEM120A, LARGE, IRS1, TBCB, BLVRB, CHST7, ALOX5AP, KCNJ8, HSPB1, TANGO2, ZNF438, ZNF438, CHCHD10, SLC35E3, ARL8A, COX17, AGPAT6, TIPARP		NXPE3, SIDT2, SLC38A11, AGPAT6, STX2, RHOQP2, BCAT2, SNTB2, YBX3,CHCHD10, ATL3, C9orf91, CST3	
	**1 loci exclusively in “P4 vs. Vh”**		**42 loci exclusively in “E2P4 vs. Vh”**		**1 loci exclusively in “MPA vs. Vh”**		**18 loci exclusively in “E2MPA vs. Vh”**		**17 loci exclusively in “LNG vs. Vh”**		**8 loci exclusively in “E2LNG vs. Vh”**		**67 loci exclusively in “NETA vs. Vh”**		**4 loci exclusively in “E2NETA vs. Vh”**	
**Down-regulated Genes**	ZNF704		BIVM, ORC2, NPAT, CNOT6L|CNOT6LP1, NQO1, LRRC37A4P, CREB5, ZNF462, SMAD5, DLC1, GRIA3, SMC5, CACNA2D1, C14orf1, NUCKS1, NEFM, PLEKHA5 TNFRSF10B, CNN3, NEO1, CDK19, HNRNPA1, LPHN1, CDH11, MXRA5P1 MASP1, HNRNPA1, TFAM, HNRNPA1, PPP1CC, ZKSCAN8, COL6A3, TFDP2, TSPYL2, LRRC8C, FAM171B, TLE4, CH25H, TMEM97, SLC7A11, PCDH18		FAM46C		MACF1, FAM46C, CACNA1C, ABCG1, FHL3, NF1P3, PTMAP4, TMEM51, PMEPA1, ARL15, CNNM1, RPL22L1, BAZ2B, PTMAP4, ZNF33B, NES, TMEM25, CADM4		NHSL2, FAM43A, ALDH1A1, PARD3, RASL11A, ANGPT1, PCYT1B, DEPDC7, NPY1R, CKS1B, MBIP, FIGNL2, CADM4, PHLDA1, WBP1L, LOXL2, ACSL4		PCNXL4, GABPA, TNRC6B, PCMTD2, CDK2, SNRK, STRA6, CEP57		IFI16, PTMAP4, CROT, TOP2B, SYDE2, IRF1, CNN2, PAK1, RHBDD2, FARP1, CDK2, PCNXL4, CNOT2, ZNF483, CNNM1, ATP1A1, TANC1, TNFRSF10B, MACF1, CCDC109B, PTPN14, TGFBRAP1, HMGN1P30, TRIM5, CREB5, TFAM, PTDSS1, PPP1CC, TERF1, HNRNPA1, HNRNPA1P10, RNU6-674P, DUSP7, CH25H, TSHZ1, METAP1, ARL15, HNRNPA1P7, TBC1D8, RUNX1T1, ESR1, SALL2, PNISR, DGKH, HNRNPA1P6, EPHA5, NEO1, CCDC125, BAZ2B, ZNF713, C21orf91, CEP57, ZNF721, PDGFD, ADCY4, ZNF286A, IL17RD, SMC5 GABPA, FZD6, RBFOX2, DTWD1, PCDH18, RNU6-1152P, ZKSCAN8, LINC00597, ZNF217		ZNF483, PTMA, PDE9A, TM7SF2	

E_2_: estradiol; P_4_: progesterone; MPA: medroxyprogesterone acetate; LNG: levonorgestrel; NETA: norethindrone acetate; Vh: vehicle; ↓, Decreased; ↑, Increased.

**Table 5:** Common biofunctions and associated differentially expressed genes between progestins without and with addition of E_2_. E_2_: estradiol; P_4_: progesterone; MPA: medroxyprogesterone acetate; LNG: levonorgestrel; NETA: norethindrone acetate; Vh: vehicle; DEG: differentially expressed genes.

	Common Functions between Progesterone and Testosterone Structurally Related Progestins (Predicted Activation, z-score≥2)	Common DEG Progestins Related to Progesterone (P_4_/MPA)	Common DEG Progestins Related to Testosterone (LNG/NETA)
**−E2**	**Cell viability of tumor cell lines (Decreased)**	ABCA1, AK5, APOE, **CCL2**, CD200, DUSP6, FKBP5, GUCY1A3, INSR, KRT19, MAPK3K4, NTF3, SMAD3, TOX, ZBTB16	AK5, AKAP13, APOE, ASAH1, BCL6, BDNF, BEX2, CASP1, CAV1, **CCL2**, CCND1, CD200, CDH2, CTGF, CXCL12, DNM1, DUSP6, FGFR1, FKBP5, GDF15, GUCY1A3, HMOX1, ID4, IL6, INPP5A, INSR, IRS2, JMJD1C, KIT, KRT19, LIMK2, MAP3K4, MCOLN1, NFAT5, NR1D1, NRP1, NTF3, PGRMC1, PIK3R1, PLAU, PLK2, POLB, PTPN11, PTPRK, SMAD3, TOX, TRIB2, UGCG, VEGFA, WEE1, ZBTB16
**+E2**	**Differentiation of fibroblasts (Decreased)**	**CCL2**, CD44, DKK1, F2RL1, **IL6**, PDE5A	**CCL2**, DKK1, F2RL1, **IL6**, PDE5A
	**Growth of connective tissue (Decreased)**	**CCL2**, CCND1, CD44, CDH13, CTGF, CXCL12, FGF1, FGF7,, FGF9, FOXO1, GREM1, IGFBP4, **IL6**, PDE5A, PDGFD, PLAU, PTPRK, RUNX1T1, SFRP1, SMAD3, SPRY2, THRB, TMPO, WNT2	**CCL2**, CCND1, CD83, CDH13, CTGF, CXCL12, FGF1, FGF7, FGF9, FGFR1, FOXO1, GREM1, IGFBP4, **IL6**, PDE5A, PDGFC, PLAU, PRDX4, PTPRK, RUNX1T1, SFRP1, SMAD3, SPRY2, THRB, TMPO, WNT2
	**Invasion of cells (Decreased)**	ABLIM1, BDKRB1, BDNF, CAV1, **CCL2**, CCND1, CD44, CDH13, CDH2, CFH, CNR1, CTGF, TSB, CTSH, CTSL, CXCL12, DKK1, DOCK4, DRAM1, DUSP6, EFNB3, ELMO1, ETV1, ETV4, ETV5, FHL2, FUT8, GDF15, HMOX1, HTRA1, **IL6**, IRS2, JUNB, JUP, KIT, KRT19, LCP1, LIMA1, LIMK2, LPAR1, MGAT5, MMP16, MMP3, MSI2, NFAT5, NRP1, NRP2, PDLIM1, PIK3R1, PLAU, PTPRK, RECK, RGS4, SATB1, SEMA5A, SERPINE1, SFRP1, SMAD3, SPARCL1, SPRY2, SPSB1, TCF4, TFAP2C	ACSL4, BDKRB1, BDNF, CAV1, **CCL2**, CCND1, CDH13, CDH2, CFH, CNR1, CTGF, CTSB, CTSH, CTSL, CXCL12, DIAPH2, DKK1, DOCK4, DPYSL3, DRAM1, DUSP6, EFNB3, ELMO1, ESR1, ETV1, ETV4, ETV5, FGFR1, FHL2, FUT8, GDF15, HMOX1, HTRA1, ID4, **IL6**, IRS2, JUNB, JUP, KIT, KRT19, LCP1, LIMA1, LIMK2, LOXL2, LPAR1, MGAT5, MMP3, MSI2, NFAT5, NRP1, NRP2, PDLIM1, PGR, PIK3R1, PLAU, PTPRK, RECK, RGS4, SATB1, SEMA5A, SERPINE1, SFRP1, SMAD3, SPARCL1, SPRY2, SPSB1, TCF4, TFAP2C, TGFB3, TGFBI, TMPO, VEGFA, ZBTB16
	**Invasion of tumor cell lines (Decreased)**	ABLIM1, BDKRB1, BDNF, CAV1, **CCL2**, CCND1, CD44, CDH13, CDH2, CFH, CNR1, CTGF, CTSB, CTSH, CXCL12, DKK1, DOCK4, DRAM1, DUSP6, EFNB3, ELMO1, ETV1, ETV4, ETV5, FHL2, FUT8, GDF15, HMOX1, HTRA1, **IL6**, IRS2, JUNB, KIT, KRT19, LCP1, LIMA1, LIMK2, LPAR1, MGAT5, MMP16, MMP3, MSI2, NFAT5, NRP1, NRP2, PDLIM1, PIK3R1, PLAU, PTPRK, RECK, SATB1, SEMA5A, SFRP1, SMAD3, SPARCL1, SPRY2, SPSB1, TCF4	ACSL4, BDKRB1, BDNF, CAV1, **CCL2**, CCND1, CDH13, CDH2, CFH, CNR1, CTGF, CTSB, CTSH, CXCL12, DIAPH2, DKK1, DOCK4, DPYSL3, DRAM1, DUSP6, EFNB3, ELMO1, ESR1, ETV1, ETV4, ETV5, FGFR1, FHL2, FUT8, GDF15, HMOX1, HTRA1, ID4, **IL6**, IRS2, JUNB, KIT, KRT19, LCP1, LIMA1, LOXL2, LPAR1, MGAT5, MMP3, MSI2, NFAT5, NRP1, NRP2, PDLIM1, PGR, PIK3R1, PLAU, PTPRK, RECK, SATB1, SEMA5A, SFRP1, SMAD3, SPARCL1, SPRY2, SPSB1, TCF4, TFAP2C, TGFB3, TGFBI, TMPO, VEGFA
	**Migration of breast cancer cell lines (Decreased)**	**CCL2**, CCND1, CD44, CDH2, CTGF, CTSL, CXCL12, DUSP6, FGF1, FGF7, GAB1, **IL6**, ITGA6, KRT19, NRP1, PIK3R1, PLAU, SEMA3C, SERPINE1, SMAD3, TFAP2C, TGFB3, THRB, TNFRSF21	ACSL4, **CCL2**, CCND1, CDH2, CTGF, CTSL, CXCL12, DUSP6, ESR1, FGF1, FGF7, GAB1, **IL6**, KRT19, NRP1, PGR, PIK3R1, PLAU, SEMA3C, SERPINE1, SLC16A4, SMAD3, TFAP2C, TGFB3, THRB, TNFRSF21, VEGFA
	**Migration of tumor cell lines (Decreased)**	BDKRB1, BDNF, CAV1, **CCL2**, CCND1, CD44, CDH13, CDH2, CTGF, CTSB, CTSH, CTSL, CXCL12, DUSP6, EFNB3, ELMO1, ETV4, ETV5, F2RL1, F3, FGF1, FGF7, FHL2, GAB1, GDF15, HMOX1, HTRA1, GFBP4, **IL6**, ITGA6, ITPR1, JUP, KDR, KIT, KRT19, LIMK2, LPAR1, LTBP2, MGAT5, MME, MMP19, MMP3, MYO10, NRP1, NRP2, NTF3, PDLIM1, PEAK1, PIK3R1, PLAU, PLCL1, PTGER4, PTPN11, PTPRK, RAP2A, RHOU, SEMA3C, SEMA5A, SERPINE1, SMAD3, SPARCL1, SPRY2, SPSB1, TCF4, TFAP2C, TGFB3, TGFBI, THRB, TMPO, TNFRSF21	ACSL4, BDKRB1, BDNF, CAV1, **CCL2**, CCND1, CDH13, CDH2, CTGF, CTSB, CTSH, CTSL, CXCL12, DUSP6, EFNB3, ELMO1, ESR1, ETV4, ETV5, F2RL1, F3, FGF1, FGF7, FGFR1, FHL2, GAB1, GDF15, HMOX1, HTRA1, IGFBP4, **IL6**, ITPR1, JUP, KIT, KRT19, LPAR1, LTBP2, MGAT5, MME, MMP19, MMP3, MYO10, NRP1, NRP2, NTF3, PDLIM1, PEAK1, PGR, PIK3R1, PLAU, PLCL1, PTGER4, PTPN11, PTPRK, RAP2A, RHOU, SEMA3C, SEMA5A, SERPINE1, SH3PXD2B, SLC16A4, SMAD3, SPARCL1, SPRY2, SPSB1, TBX3, TCF4, TFAP2C, TGFB3, TGFBI, THRB, TMPO, TNFRSF21, VEGFA

**Table 6:** Fold change of common DEG in all progestins in absence or presence of E_2_. E_2_: estradiol; P_4_: progesterone; MPA: medroxyprogesterone acetate; LNG: levonorgestrel; NETA: norethindrone acetate; Vh: vehicle.

	Progestins Related to Progesterone (P4/MPA)				Progestins Related to Testosterone (LNG/NETA)			
	P_4_ vs. Vh	E_2_P_4_ vs. Vh	MPA vs. Vh	E_2_MPA vs. Vh	LNG vs. Vh	E_2_LNG vs. Vh	NETA vs. Vh	E_2_NETA vs. Vh
**CCL2**	−1.55	−7.44	−4.77	−6.04	−6.41	−6.25	−6.16	−5.08
**IL6**		−2.74	−1.93	−2.53	−2.82	−2.93	−2.92	−2.83
**VEGFA**		−1.76			−1.68	−1.78	−1.74	−1.51

## Supplementary Materials

Supplementary materials can be found at https://www.mdpi.com/1422-0067/21/7/2625/s1. Supplemental Table S1. Differentially expressed genes of each progestin vs. vehicle without and with addition of E_2_ (FC ≥ 1.5). Highlighted rows are the common genes between each progestin without or with addition of estradiol. E_2_: estradiol; pink: progesterone (P_4_); green: medroxyprogesterone acetate (MPA); blue: levonorgestrel (LNG); yellow: norethindrone acetate (NETA); FC: fold change. Supplemental Figure S1. IGFBP1 protein level in different treatments. Protein levels of IGFBP1, a progesterone target gene, was determined by ELISA in conditioned media of *n*=5 eSF cell lines grown in culture and normalized to cell number. For each sample, each assay was done in duplicate. Values for each progestin or progestin plus E_2_ is the measurements from 5 samples with error bars.
