# Supplementary figures and images for: Progestins Related to Progesterone and Testosterone Elicit Divergent Human Endometrial Transcriptomes and Biofunctions
Source: Int J Mol Sci. 2020 Apr 9;21(7):2625. doi: 10.3390/ijms21072625 (PMC7177488; doi:10.3390/ijms21072625)

**Supplementary Table 1. IGFBP1 Protein level in different treatments.**

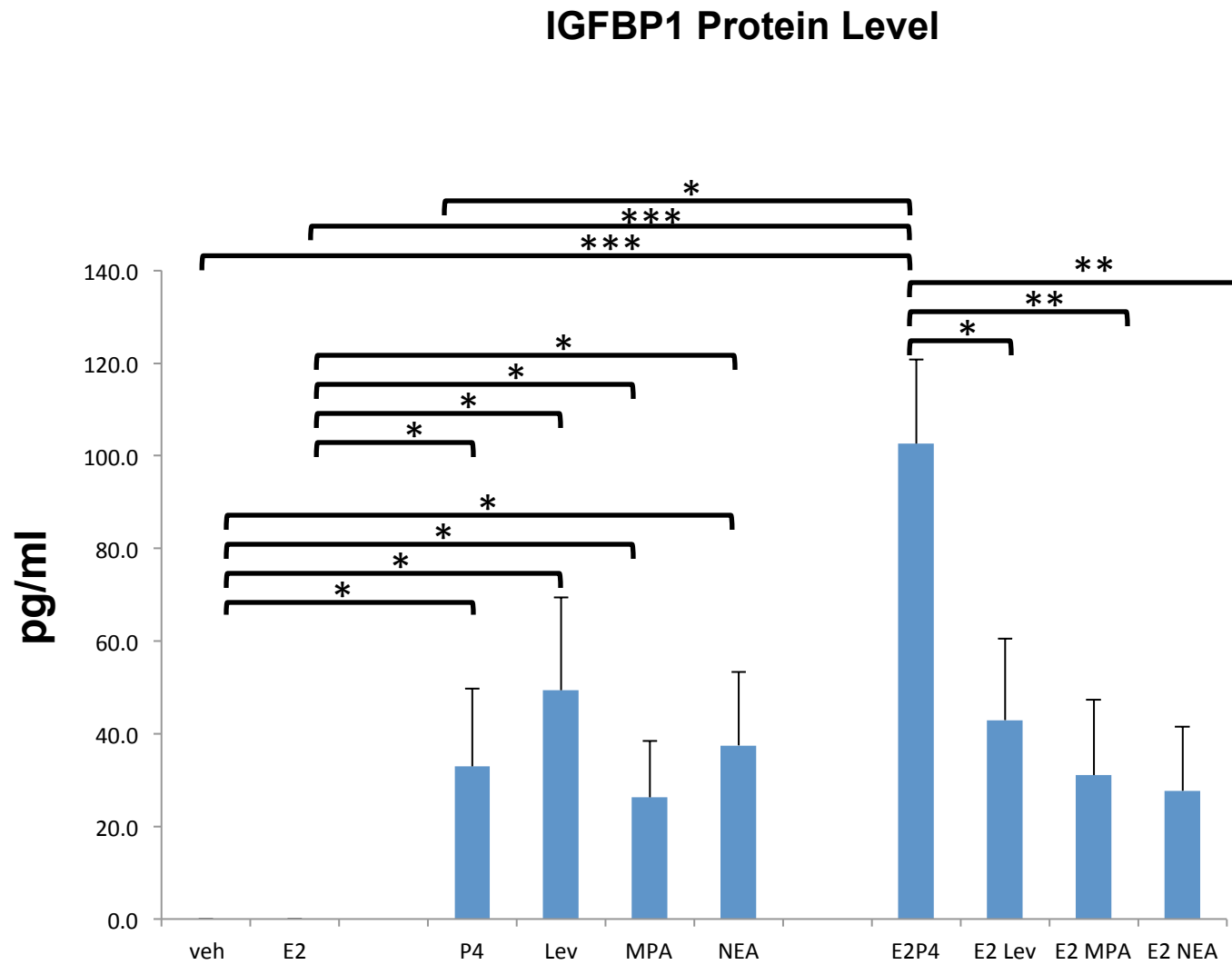

Supplement: Supplementary file 1 [file ijms-21-02625-s001.pdf]
